# The Validation of a Pocket Worn Activity Tracker for Step Count and Physical Behavior in Older Adults during Simulated Activities of Daily Living

**DOI:** 10.1177/2333721420951732

**Published:** 2020-09-30

**Authors:** Darcy Ummels, Wouter Bijnens, Jos Aarts, Kenneth Meijer, Anna J. Beurskens, Emmylou Beekman

**Affiliations:** 1 Research Centre for Autonomy and Participation of Persons with a Chronic Illness, Zuyd University of Applied Sciences, Heerlen, Netherlands; 2 Department of Family Medicine, CAPHRI School for Public Health and Primary Care, Maastricht University Medical Centre, Maastricht, Netherlands; 3 ParaMedisch Centrum Zuid, Sittard, Netherlands; 4 Instrument Development, Engineering and Evaluation, Maastricht University, Maastricht, Netherlands; 5 Department of Nutrition and Movement Sciences, NUTRIM school for Nutrition and Translational Research in Metabolism, Maastricht University, Maastricht, Netherlands

**Keywords:** Wearable, step count, physical activity, validation, older adults, ADL

## Abstract

**Purpose:**

The purpose of this study was to validate optimized algorithm parameter settings for step count and physical behavior for a pocket worn activity tracker in older adults during ADL. Secondly, for a more relevant interpretation of the results, the performance of the optimized algorithm was compared to three reference applications

**Methods:**

In a cross-sectional validation study, 20 older adults performed an activity protocol based on ADL with MOX_MissActivity_ versus MOX_Annegarn_, activPAL, and Fitbit. The protocol was video recorded and analyzed for step count and dynamic, standing, and sedentary time. Validity was assessed by percentage error (PE), absolute percentage error (APE), Bland-Altman plots and correlation coefficients.

**Results:**

For step count, the optimized algorithm had a mean APE of 9.3% and a correlation coefficient of 0.88. The mean APE values of dynamic, standing, and sedentary time were 15.9%, 19.9%, and 9.6%, respectively. The correlation coefficients were 0.55, 0.91, and 0.92, respectively. Three reference applications showed higher errors and lower correlations for all outcome variables.

**Conclusion:**

This study showed that the optimized algorithm parameter settings can more validly estimate step count and physical behavior in older adults wearing an activity tracker in the trouser pocket during ADL compared to reference applications.

## Introduction

In the past decade, activity trackers have been used more frequently by a relatively young and physically active population (Macridis et al., 2018). In addition to this population, activity trackers can also be beneficial for older adults (65+). In 2018, only 37% of the older adults in the Netherlands were sufficiently physically active according to Dutch guidelines (National Institute for Public Health and the Environment, 2015). Activity trackers can contribute to overcome this by giving insight into the amount of physical activity, increasing awareness and motivating older adults to be more physically active (Maher et al., 2017; Mercer et al., 2016; O’Brien et al., 2015; Preusse et al., 2017; Sullivan & Lachman, 2016; Ummels et al., 2019)

Several studies have shown that older adults are most interested in step count and amount of physical behavior as outcome variables for physical activity (Maher et al., 2017; Rosenberg et al., 2016; Schlomann, 2017; Ummels et al., 2019). Recent studies have shown that step count and physical behavior are not validly measured by consumer-grade activity trackers during low walking speeds, which often occur during activities of daily living (ADL) such as household activities (Alharbi et al., 2016; Beevi et al., 2016; Cyarto et al., 2004; Evenson et al., 2015; Ferguson et al., 2015; Floegel et al., 2016; Martin et al.,2012; Straiton et al., 2018; Tedesco et al., 2019; Ummels et al., 2018; Van Blarigan et al., 2017). This lower validity can partly be explained by the fact that the majority of consumer-grade activity trackers don’t have older adults as a target group and don’t adjust their algorithms accordingly.

Recently, an adjustable classification algorithm was published to optimize algorithm performance (Bijnens et al., 2019). Through easily adjustable algorithm parameters it is possible to optimize the performance of this algorithm for different target and tracker wear locations. A recent qualitative study showed that older adults would prefer to wear an activity tracker in their trouser pocket (Ummels et al., 2019). Consequently, the adjustable algorithm was optimized to estimate step count and dynamic, standing, and sedentary time for older adults and a pocket worn activity tracker according to the proposed method by Bijnens et al. (2019).

The first purpose of this study was to validate these optimized algorithm parameter settings for step count and physical behavior expressed as dynamic, standing, and sedentary time in older adults with a normal pattern wearing an activity tracker in their trouser pocket during simulated ADL. Secondly, to have a more relevant interpretation of the validation results, the performance of the optimized algorithm parameter settings for older adults was compared to the algorithm where the adjustable classification algorithm originates from and two frequently used activity trackers.

## Methods

### Study Design

A cross-sectional validation study was performed in which the optimized algorithm parameter settings were validated and compared to the algorithm where the adjustable classification algorithm originates from and two activity trackers.

### Participants

Twenty older adults were recruited from several local associations for older adults (e.g., bridge club or church association) in the South of the Netherlands. Participants were included if they were older than 65 years and didn’t meet the Dutch physical activity guidelines (a minimum of 150 min of moderate-intensity per week (Health Council of the Netherlands, 2017)). Exclusion criteria were insufficient understanding of the Dutch language, use of a walking aid, and a pathological gait during the 10-metre walk test (10MWT) observed by a physiotherapist (Collen et al., 1990). All participants provided written informed consent prior to inclusion.

### Activity Protocol

A participant-determined sequence activity protocol was developed based on ADL. To simulate free-living, participants were free to choose the order and duration of a series of daily activities. The activity protocol, shown in Table 1, was based on earlier activity protocols with ADL in people with chronic diseases and older adults (Cavalheri et al., 2011; Erasmus MC University Medical Center Rotterdam, 2013; Langer et al., 2009; Sant’Anna et al., 2012; Ummels et al., 2018).

**Table 1.** The Participant-Determined Sequence Activity Protocol with Activities of Daily Living for Older adults.

Activity type	Defined for the gold standard as
Squat^a^	Marks start of the protocol
Organising a cabinet with cutlery, plates, and cups	Standing
Reading the newspaper while seated at a table	Sedentary
Ironing and folding laundry	Standing
Sitting and talking	Sedentary
Washing the dishes	Standing
Sweeping the floor	Dynamic
Changing linens on a bed	Dynamic
Setting the table with cutlery, plates, and cups	Dynamic
Squat^a^	Marks end of the protocol

*Note*. ^a^Squat was mandatory at the beginning and at the end of the activity protocol and was not used for analysis.

### Activity Trackers

**Figure 1.**
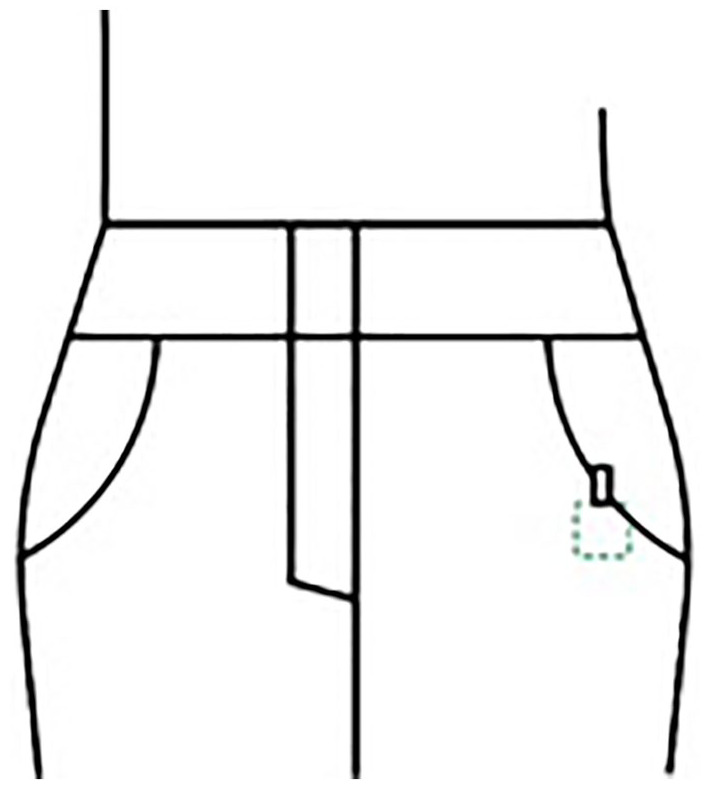
MOX wear location.

The MOX Activity Logger (MOX; Maastricht Instruments, Maastricht, NL) (Maastricht Instruments BVa, 2020) contains a tri-axial accelerometer (ADXL362, Analog Devices, Norwood, MA, US). This small, light-weight, waterproof device (35 mm × 35 mm × 10 mm, 11 g) measures raw acceleration data (±8 g) in three orthogonal sensor axes (X, Y, and Z) at a 25 Hz sampling rate. The raw data is stored directly on the internal memory. The MOX has storage capacity and battery life for continuous measurements up to 7 days. Device configuration, data transfer and charging of the device are possible via an USB connection. Data analysis is performed offline. The MOX was worn in the front trouser pocket, attached with a clip, to secure a fixed orientation of the device with respect to axial mobility of the upper leg. This wear location is shown in Figure 1.

Two commonly used activity trackers, the activPAL (activPAL3, PAL Technologies Ltd, Glasgow, Scotland, UK) and the Fitbit Alta HR (Fitbit; Fitbit Inc., San Fransisco, CA, US) were used as reference for a more relevant interpretation of the validation results. Based on the recommendations of the manufactures the activPAL is worn on the dominant leg ten centimetres above the patella (activPAL) and the Fitbit is worn on the non-dominant wrist (Fitbit, 2020a).

### Data Collection and Procedure

Participants were measured at the Human Performance Laboratory of Maastricht University (Maastricht, NL) or at Zuyd University of Applied Science (Heerlen, NL). Both laboratories are comparable in size (about 120 m^2^) and facilities. Demographic data were collected (gender, age, body weight, and body length) by two researchers, either DU (physiotherapist) or WB (application engineer). Thereafter, the participants performed the 10 MWT to calculate their average walking speed. After the 10 MWT, participants were fitted with the MOX, the activPAL, and the Fitbit. The same MOX, activPAL, and Fitbit were used for all participants.

The activity protocol was recorded on video and observed to use as a gold standard to determine the actual step count, dynamic, standing, and sedentary time in seconds performed by the participants. Step count was counted manually by two independent observers using the counter application Counter+ (Seedform, 2020). A step was defined as: “when the entire foot is lifted from the floor and when the participants replaced their foot (forward, backward, sideways or upwards)” (Beekman et al., 2017). After manually counting the step count, the video was re-observed and the time that the participants performed dynamic (walking and walking during household activities), standing or sedentary (sitting, lying) time was noted. Physical behavior was assessed by two independent observers (Table 1) using the EasyTag app (Dartfish Ltd, 2020).

The data from the activity trackers were collected directly after the activity protocol. Analysis of the raw acceleration data of the MOX took place on a PC after the measurements (off-line) using Matlab (R2018b, The MathWorks Inc., Natick, MA, US) with two algorithms. The first one is the activity classification algorithm presented and validated by Annegarn et al. (2011) for healthy adults (MOX_Annegarn_), where the adjustable classification algorithm originates from. The second one is the classification algorithm with application specific adjustable parameters itself (Bijnens et al., (2019). For application in an older adult target group wearing an activity tracker in their trouser pocket the optimized parameter settings are: a data segmentation window size of 2 s, an amount of physical activity threshold of five counts per second (cps) and an orientation threshold of 0.8 g. This application is referred to as Miss Activity, the parameter settings as MOX_MissActivity_. For MOX_Annegarn_ dynamic, standing, and sedentary time spent in seconds were retrieved. In addition to these three variables, for MOX_MissActivity_ step count was also retrieved. For the activPAL, step count and dynamic, standing, and sedentary time spent in seconds were retrieved from the PAL Software Suite (v7.2.32; PAL Technologies Ltd., Glasgow, Scotland, UK). For the Fitbit, step count and active minutes (by definition: ten continuous minutes long bouts of moderate-to intense activity >3 metabolic equivalent of task [MET]) (Fitbit Inc, 2020b), were retrieved from the corresponding Fitbit app (Fitbit Inc., San Fransisco, CA, US). From this point, we refer to the active minutes of the Fitbit as dynamic time.

### Data Analysis

Data analysis was performed using SPSS Statistics (version 23.0; IBM Corp, Armonk, NY, US) and Prism (GraphPad Prism 8.2.1(441); GraphPad Software, San Diego, CA, USA).

Descriptive statistics of the participant characteristics were presented as a number (percentage) for the categorical variable gender and as a mean (95% confidence interval [CI]) for the continuous variables age, body length, body weight, and average walk speed.

#### Inter-observer reliability of the video observations

The differences in step count, dynamic, standing, and sedentary time was calculated between two observers. If there was more than a 5% difference between the two observers, a third observer assessed the video. The inter-observer reliability of the two observers with the smallest difference was assessed by an Intraclass Correlation Coefficient (absolute agreement, two-way random) and Bland-Altman plots with limits of agreement. It was hypothesized that there was a strong correlation between observers (*r* ≥ .90) in order to guarantee a robust gold standard (De Vet et al., 2011).

#### Validation

To check for outliers in the data of MOX_MissActivity_, MOX_Annegarn_, activPAL, Fitbit versus the video observations regarding the variables step count, dynamic, standing, and sedentary time the data were transformed to z-scores and Bland-Altman plots were visually inspected. In case of outliers, pairwise deletion was applied.

For step count, dynamic, standing, and sedentary time the mean (95% CI), mean difference, the percentage error (PE), the absolute percentage error (APE), and the smallest detectable change (SDC) were used to gain insight into the algorithm’s and activity trackers’ performance compared to video observations.

(1) $$PE=\frac{\begin{array}{l} Variable_{Gold\;standard}-\\ Variable_{Actvity\;tracker} \end{array}}{Variable_{Gold\;standard}}*100$$

(2) $$APE=\frac{\left|\begin{array}{l} Variable_{Gold\;standard}-\\ Variable_{Activity\;tracker} \end{array}\right|}{Variable_{Gold\;standard}}*100$$

(3) $$SDC=1.96*\sqrt{2}*standard\;error\;measurment$$

Formula 1 and 2 show the calculation of PE and APE for each variable. A PE or APE of less than 10% was considered acceptable (Sasaki et al., 2016). Formula 3 shows the calculation of SDC.

The level of agreement between step count, dynamic, standing, and sedentary time and the video observations were examined by a Bland-Altman plot with their limits of agreement (Bland & Altman, 1986 ). Pearson correlation coefficients were calculated to gain insight into the relationship between the MOX_MissActivity_, MOX_Annegarn_, activPAL, Fitbit versus the video observations regarding the variables step count, dynamic, standing, and sedentary time. It was hypothesised that there would be at least a substantial correlation (*r* ≥ .60) (De Vet et al., 2011). A paired sample *t*-test was used to determine large systematic differences between the MOX_MissActivity_, MOX_Annegarn_, activPAL, Fitbit versus the video observations regarding the variables step count, dynamic, standing, and sedentary time. A *p*-value below .05 was considered to be statistically significant. Additionally the sensitivity, specificity and accuracy are calculated.

## Results

### Participant Characteristics

Twenty older adults were recruited for this study. The participant characteristics are displayed in Table 2.

**Table 2.** Participant Characteristics.

Characteristic	Participants (*n* = 20)
Gender, male, *n* (%)	10 (50%)
Age, years, mean (95% CI)	74.5 (70.9–77.6)
Body weight, kilograms, mean (95% CI)^a^	86.1 (73.7–98.5)
Body length, centimetres, mean (95% CI)	172.5 (167.6–176.9)
Average walk speed, mean (95% CI)	1.1 (1.0–1.2)

*Note*. ^a^There was one (5%) missing value for body weight.

### Inter-Observer Reliability of the Video Observations

The inter-observer reliability of the video observations calculated for step count was high (ICCagreement 0.98, *P* < .001 95% CI 0.95–0.99). The inter-observer reliability of dynamic, standing, and sedentary time were also high (ICCagreement 0.98, *P* < .001, 95% CI 0.95–0.99), (ICCagreement 0.99, *P* < .001 95% CI 0.98–0.99), (ICCagreement, 1.0, *P* < .001, 95% CI 0.99–1.0) respectively. The limits of agreement for step count (−58 to 62 steps), dynamic (−49 to 41 s), standing (−49 to 40 s) and sedentary time (−26 to 28 s) showed no systematic differences. A third observer had to be included in two cases.

### Step Count

**Figure 2.**
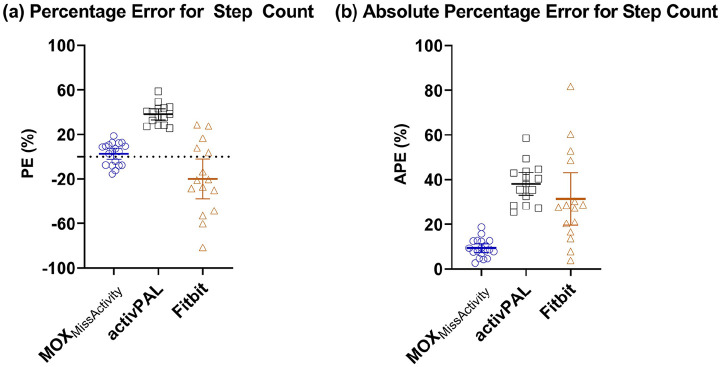
(a) Percentage error and (b) absolute percentage error for step count. Step count for the MOX_MissActivity_ is presented in blue, for activPAL in black and for Fitbit in brown.

**Figure 3.**
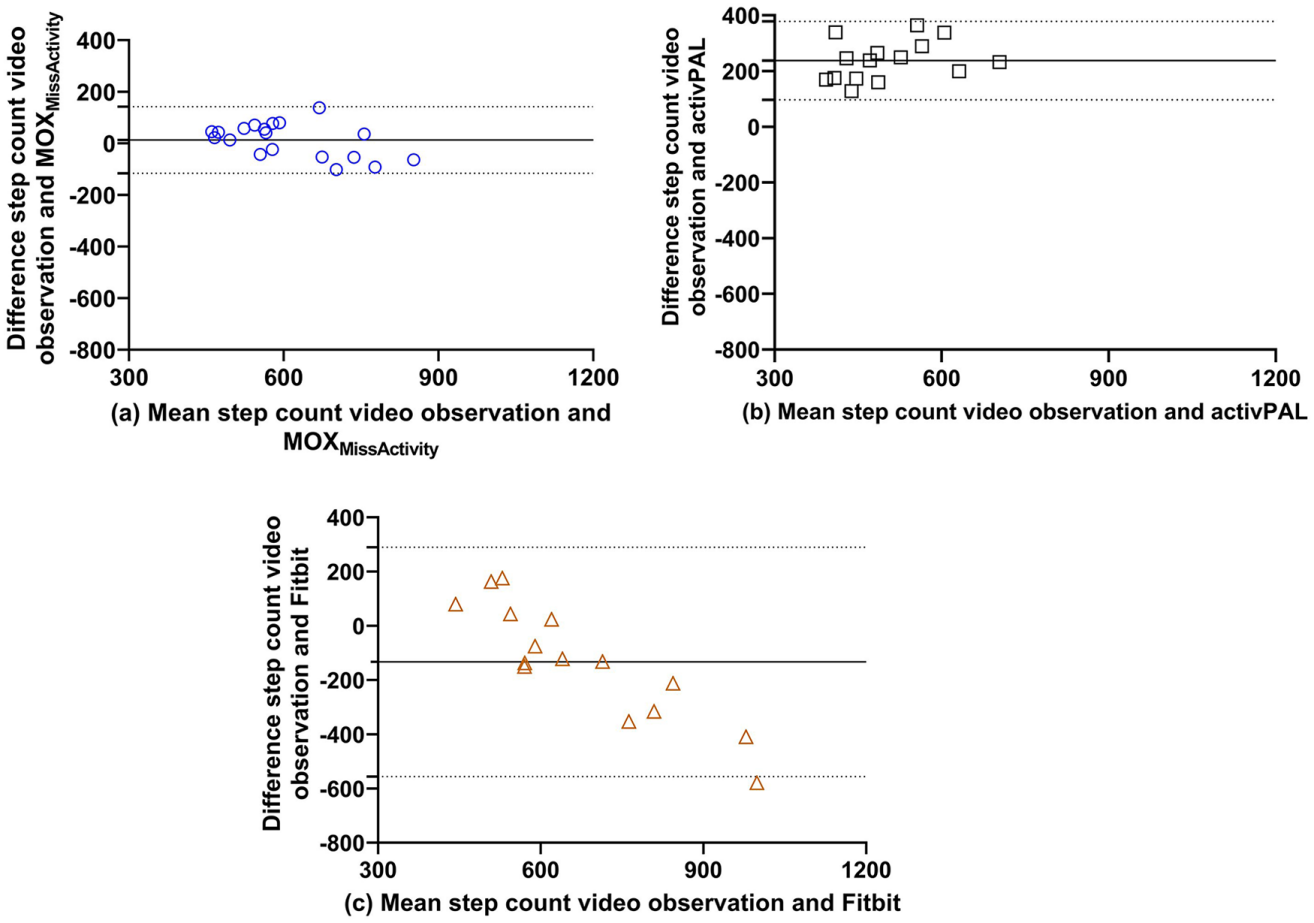
Bland-Altman plots of the (a) MOX_MissActivity_, (b) activPAL, and (c) the Fitbit versus the video observation.

Descriptive statistics for each activity tracker are shown in Table 3. The mean step count during the activity protocol counted by the video observation was 615 (566–664) steps. The MOX_MissActivity_ had a mean step count of 602 (537–667) steps, the activPAL had a mean step count of 385 (336–433) steps and the Fitbit had a mean step count of 731 (590–873) steps. The values of the percentage error and the absolute percentage error are presented in Figure 2. The Bland-Altman plots (Figure 3) show a slight overestimation of the number of steps in the MOX_MissActivity_, an overestimation in the activPAL and underestimation in the Fitbit. If the limits of agreement for the activPAL and Fitbit Alta HR are corrected for their respective bias they are −141 to 140 and −183 to 183, respectively.

**Table 3.** Descriptive Statistics, Pearson Correlation Coefficient and Paired Sample *t*-Test of Step Count by the MOX_MissActivity_ Compared to the Video Observations in Comparison with Reference Applications.

Activity tracker	Difference in step count^a^, mean (95% CI)	Percentage error, mean (95% CI)	Absolute percentage error, mean (95% CI)	Limits of agreemen*t* (lower bound–upper bound)	Pearson correlation coefficient	*p*-value	Smallest detectable change (steps)
MOX_MissActivity_^b^	13 (−19 to 45)	2.6 (−2.6 to 7.4)	9.3 (7.3 to 11.3)	−116 to 142	0.88	.40	129
activPAL^c^	238 (198 to 277)	38.1 (33.0 to 43.1)	38.1 (33.0 to 43.1)	97 to 378	0.75	>.001	485
Fitbit^c^	−160 (−287 to −35)	−20.0 (−38.3 to −1.9)	31.3 (19.6 to 43.0)	−556 to 290	0.77	.16	522

*Note*. ^a^Video observation minus activity tracker.

b One missing value for step count in the MOX_MissActivity_ (1/20, 5%).

c Five missing values for step count in the activPAL and Fitbit (5/20, 25%).

### Physical behavior

The mean dynamic, standing, and sedentary time during the activity protocol counted by the video observations were 422 (387–457), 668 (579–757), and 1716 (1452–1981) seconds, respectively. On average the total protocol lasted 45 (40–51) min. For the MOX_MissActivity_ and MOX_Annegarn_, two outliers were detected for standing time, both outliers were visible in the Bland-Altman plots. The video recordings were re-watched and, in both participants, the MOX was incorrectly worn. One of these outliers was also statistically detected with the z-score (5% with a *z*-score of >2.58). One outlier for the activPAL was detected for dynamic time, the outlier was visible in the Bland-Altman plot and was detected with the z-score (5% with a z-score of >3.29).

The mean dynamic, standing, and sedentary time for the MOX_MissActivity_ without outliers was 405 (338–473), 696 (504–889), and 1692 (1383–2001) seconds, respectively. For the MOX_Annegarn_ the mean dynamic, standing, and sedentary time was 152 (131–174), 927 (712–1141), and 1715 (1392–2038), respectively. For the activPAL, the mean dynamic, standing, and sedentary time was 309 (255–364), 1256 (859–1645), and 1234 (946–1522) seconds respectively. The mean dynamic time for the Fitbit was 1 (0–3) minutes.

**Figure 4.**
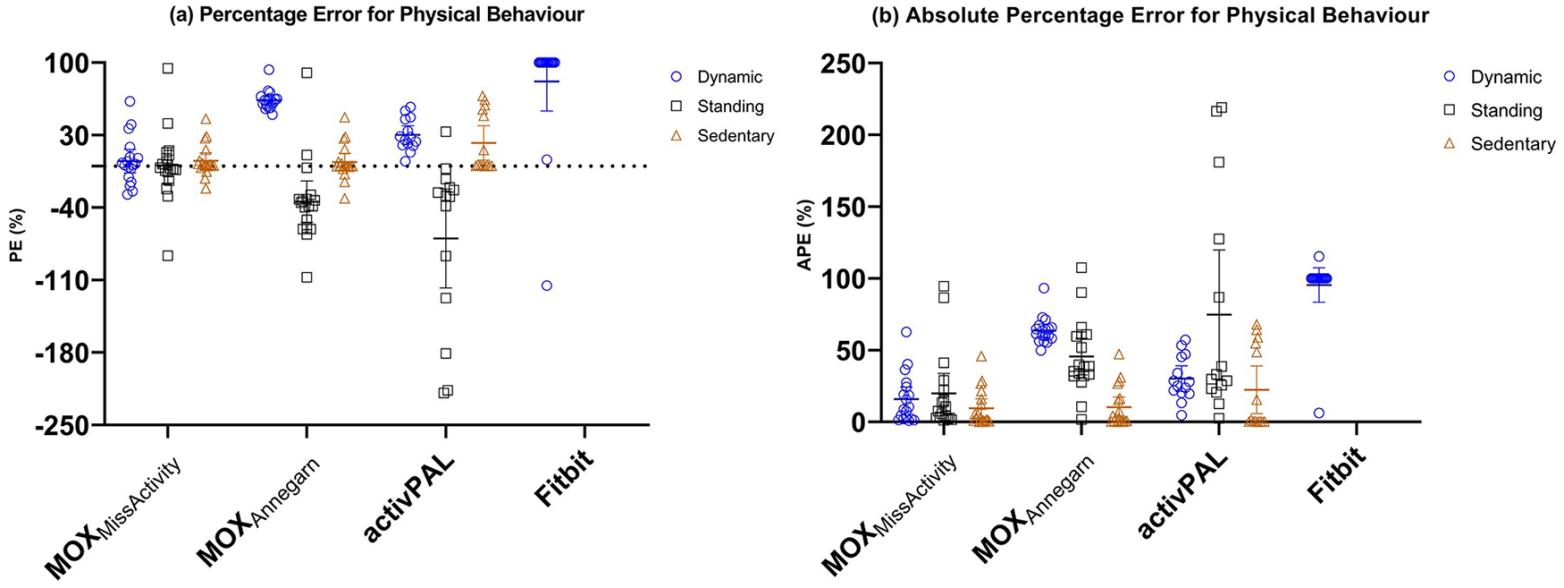
(a) Percentage Error and (b) Absolute Percentage Error for dynamic, standing, and sedentary time. PE and APE for dynamic time are presented in blue, for standing time in black and for sedentary time in brown. The Fitbit Alta HR measures dynamic time only, therefore no data for standing and sedentary time are presented.

Descriptive statistics without outliers for each activity tracker are shown in Table 4 and the descriptive statistics with outliers for each activity tracker are shown in supplementary file 1. The PE and APE for physical behavior are shown in Figure 4. To gain more insight in validity, not only based on total time but also on window-to-window basis, additional analyzes in terms of specificity, sensitivity, and accuracy were performed (supplementary file 2).

**Table 4.** Descriptive Statistics, Pearson Correlation Coefficient and Paired Sample *t*-Test of Dynamic, Standing, and Sedentary Time by MOX_MissActivity_ Compared to the Video Observations in Comparison with Reference Applications Without Outliers.

Behavior	Difference in behavior in seconds^a^, mean (95% CI)	Percentage error, mean (95% CI)	Absolute percentage error, mean (95% CI)	Limits of agreement (lower bound–upper bound)	Pearson Correlation coefficient	*p*-value	Smallest detectable change (in seconds)
**MOX_MissActivity_ Dynamic**	20 (−35 to 74)	4.8 (−6.7 to 16.2)	15.9 (7.5 to 24.3)	−197 to 237	0.55	0.513	215
**MOX_MissActivity_ Standing**	−25 (−149 to 99)	0.7 (−16.5 to 17.9)	19.9 (6.0 to 33.7)	−515 to 460	0.91	0.113	488
**MOX_MissActivity_ Sedentary**	87 (−46 to 221)	5.0 (−2.6 to 12.8)	9.6 (3.0 to 16.0)	−441 to 616	0.92	0.845	538
**MOX_Annegarn_ Dynamic**	273 (236 to 309)	63.6 (59.0 to 68.3)	63.6 (59.0 to 68.3)	127 to 410	0.36	0.000	552
**MOX_Annegarn_ Standing**	−255 (−398 to −112)	−34.3 (−54.3 to −14.4)	45.5 (32.7 to 58.3)	−1105 to 474	0.65	0.004	743
**MOX_Annegarn_ Sedentary**	64 (−88 to 215)	3.9 (−4.7 to 12.4)	10.3 (3.3 to 17.4)	−675 to 941	0.85	0.191	599
**activPAL Dynamic** ^b^	133 (89 to 176)	30.2 (21.2 to 39.1)	30.1 (21.3 to 39.0)	−17 to 283	0.52	0.001	148
**activPAL Standing** ^b^	−519 (−873 to −166)	−69.9 (−117.7 to −22.1)	74.6 (29.5 to 119.8)	−1720 to 692	0.51	0.004	1745
**activPAL Sedentary** ^b^	449 (92 to 807)	22.4 (5.8 to 39.0)	22.4 (5.8 to 39.0)	−905 to 1804	0.29	0.001	1638
**Fitbit Dynamic** ^b^	347 (226 to 467)	81.8 (53.0 to 110.7)	96.6 (85.0 to 108.5)	−2854 to 555.7	0.05	0.000	832

*Note*. ^a^Video observations minus activity tracker.

b Five (5/20 25%) missing values for the activPAL and Fitbit.

**Figure 5.**
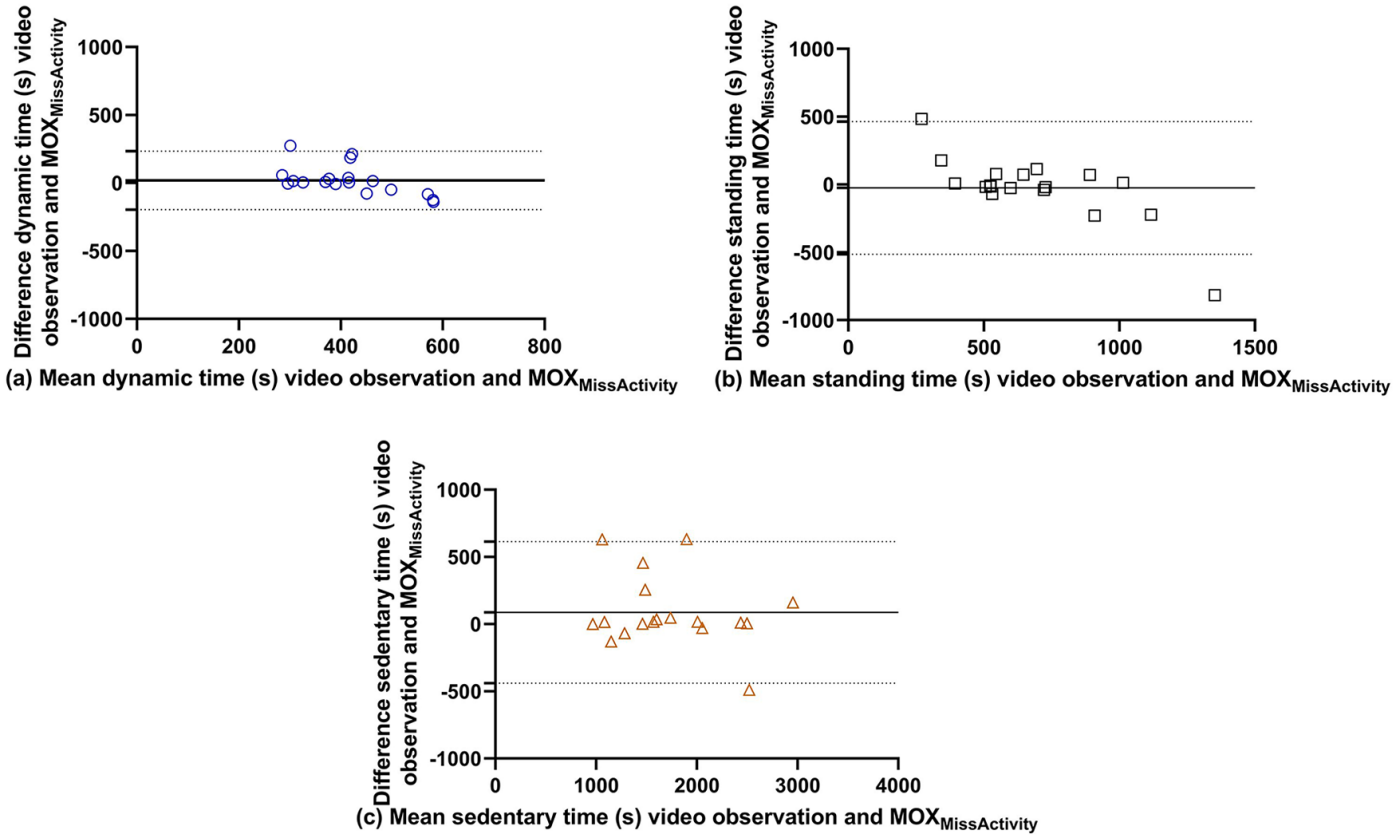
(a) Bland- Altman plots of dynamic, (b) standing, and (c) sedentary time of the MOX_MissActivity_ without outliers versus the video observations.

The Bland-Altman plots of the MOX_MissActivity_ without outliers (Figure 5) show a slight overestimation for dynamic and sedentary time and a slight underestimation for standing time. When the limits of agreement for the activPAL are corrected for their bias for dynamic, standing, and sedentary time, they are −141 to 147, −538 to 527 and −1234 to 1130 respectively.

## Discussion

### Principal Findings

This study showed that the optimized algorithm parameter settings (MOX_MissActivity_) can more validly measure step count and physical behavior expressed as dynamic, standing, and sedentary time in older adults wearing an activity tracker in their trouser pocket during ADL based on a test combination of PE, APE, correlation coefficients, and paired sample *t*-test compared to the MOX_Annegarn_, activPAL, and Fitbit.

The variables step count and sedentary time showed good validity in comparison with the gold standard. It was hypothesised that all variables of the MOX_MissActivity_ would have at least a substantial correlation (*r* ≥ .60) and an APE of <10%. Only the variable dynamic time had a slightly lower correlation coefficient of 0.55 and the variables dynamic and standing time had a mean APE of 15.9% and 19.9%. These results are supported with additional analyzes of the specificity, sensitivity, and accuracy (supplementary file 2). This can be explained by the fact that the activities in the activity protocol were classified into dynamic, standing, and sedentary behavior as a whole. For example, by definition, the video observations classified changing linens as dynamic behavior, however during this activity it is possible that the participant performed a sequence of standing and dynamic behavior (e.g., standing still to put the cushion on the bed).

In the current study, MOX_Annegarn_, activPAL, and Fitbit showed lower validity compared to the gold standard and the MOX_MissActivity_. It is clear that these target group and wear location specific classification algorithms cannot be applied outside of their specific context. The results for the activPAL are in line with a recent study showing a low validity during short stepping bouts and activities with low walking speeds, such as shuffling, picking, transitions, and kneeling in older adults (Bourke et al., 2019; Feehan et al., 2018). The underestimation for dynamic time of the Fitbit can be explained by the definition Fitbit uses for active minutes: 10 continuous minutes long bouts of moderate-to intense activity >3 MET. It is reasonable to assume that activities of daily living weren’t performed with such intensity and/or for that long during this protocol.

#### Limitations and strengths

This study had some limitations, the first one being the relatively low sample size (*n* = 20). Future work could include a larger sample size, although several validity studies have been performed with a sample size of 20 (Evenson et al., 2015). Second, that due to some technical errors, there were five missing values for both the Fitbit and the activPAL. However, since these activity trackers were only used as reference this should not have affected the main purpose of the current study. Third, the varying MOX wear location relative to the body. To secure the validity of the algorithm the MOX should always be correctly placed in the trouser pocket below the waist, this should be addressed in a manual. By re-observing the video recordings, it was noted that the MOX was placed above the participants’ hip in two cases. Since the algorithm assumes a wear location on the upper leg, the MOX was not able to correctly measure within these two participants due to this misplacement. Therefore, it was chosen to handle these two cases as outliers, since the misplacement, and not the algorithm, compromised the validity. Fourth, this study was performed in a lab setting and is therefore not directly generalizable to daily life. However, with the ADL protocol, daily life was simulated as close as possible to daily life. This is in line with the proposed standardization methods of Welk et al. (2019).

A strength of this study is the use of the participant-determined sequence activity protocol to validate the algorithm. This activity protocol simulates free-living since participants were free to choose the order and duration of the activities they performed. To simulate the free-living situation as best as possible activities that are frequently performed by older adults are included in the activity protocol. Furthermore, this study follows the recommendations made by Welk et al. (2019) for validation studies in wearables: use a diverse sample, appropriate sampling of daily behavior, an appropriate criterion measure, standardised protocols and wear locations, and inclusion of reference applications. To standardise the analyzes they recommend to use relevant metrics, documenting the error and the direction of the error and to focus on equivalence (Welk et al., 2019). Another strength of this study is the high inter-observer reliability resulting in a robust gold standard (range *r* = .96–1.0).

#### Clinical implications

From previous research it is known that consumer-grade activity trackers can’t measure step count and physical behavior validly during low walking speeds, which often occurs in older adults and during ADL (Alharbi et al., 2016; Beevi et al., 2016; Cyarto et al., 2004; Evenson et al., 2015; Ferguson et al., 2015; Floegel et al., 2016; Martin et al., 2012; Straiton et al., 2018; Ummels et al., 2018; Van Blarigan et al., 2017). Apparently, daily life of older adults differs that much from the target group of these consumer-grade activity trackers that their algorithms are not sufficient for older adults. Therefore, it is important to have an algorithm optimized for the target group, wear location and their specific activities. If a consumer-grade activity tracker is used for this target group, the algorithm should ideally be personalised to the specific target group or at least bias corrections to the outcomes of the algorithm should be applied. The validity of the optimized algorithm is limited to older adults with a normal gait pattern. This study shows that an optimized algorithm is indeed more valid than general purpose activity trackers. As is shown by the smallest detectable change the optimized algorithm could also detect change in patient’s physical activity level sooner. However, this study is performed on a group level and not on an individual level. Therefore, the interpretation on an individual level must be performed carefully since the optimized algorithm can both over- and underestimate step count and physical behavior depending on the number of steps or seconds

For an activity tracker to be useful in daily life, validity is important, but feasibility is equally important. In a future feasibility study, development of a user-friendly user-interface of the MISS Activity will be addressed. The validated algorithm together with the user-interface will be called the Measure It Super Simple (MISS) Activity (Maastricht Instruments BVb, 2020).

## Conclusion

This study showed that the optimized algorithm parameter settings can more validly estimate step count, dynamic, standing, and sedentary time in older adults with a normal gait pattern wearing an activity tracker in their trouser pocket during a participant-determined sequence activity protocol with activities of daily living compared to reference applications with generic activity tracker algorithms. For future studies and clinical practice an algorithm should ideally be optimized to the target population. Future work will include the development of a target group-specific user-friendly application.

## Supplemental Material

Supplementary_file_1_v3. – Supplemental material for The Validation of a Pocket Worn Activity Tracker for Step Count and Physical Behavior in Older Adults during Simulated Activities of Daily Living Click here for additional data file. Supplemental material, Supplementary_file_1_v3. for The Validation of a Pocket Worn Activity Tracker for Step Count and Physical Behavior in Older Adults during Simulated Activities of Daily Living by Darcy Ummels, Wouter Bijnens, Jos Aarts, Kenneth Meijer, Anna J. Beurskens and Emmylou Beekman in Gerontology and Geriatric Medicine
