# Supplementary material for: The Validation of a Pocket Worn Activity Tracker for Step Count and Physical Behavior in Older Adults during Simulated Activities of Daily Living
Source: Gerontol Geriatr Med. 2020 Sep 30;6:2333721420951732. doi: 10.1177/2333721420951732 (PMC7545746; doi:10.1177/2333721420951732)
Supplement: Supplementary_file_1_v3. – Supplemental material for The Validation of a Pocket Worn Activity Tracker for Step Count and Physical Behavior in Older Adults during Simulated Activities of Daily Living [file Supplementary_file_1_v3.pdf]

Supplementary file 1. Descriptive statistics, Pearson correlation coefficient and t-test of dynamic, standing and sedentary time by $MOX_{MissActivity}$ compared to the gold standard in comparison with reference activity trackers

**Behaviour**	**Difference in behaviour in seconds[a] mean (95% CI)**	**Percentage error mean (95% CI)**	**Absolute percentage error mean (95% CI)**	**Limits of agreement (lower bound–upper bound)**	**Pearson Correlation coefficient**	**p-value**	**Smallest detectable change in seconds**
$MOX_{MissActivity}$ Dynamic	28 (-22 to 78)	6.9 (-3.8 to 17.5)	16.9 (9.2 to 24.5)	-183 to 240	0.58	0.255	211
$MOX_{MissActivity}$ Standing	-134 (-328 to 60)	-17.3 (-48.2 to 13.6)	35.9 (8.9 to 62.8)	-947 to 679	0.76	0.370	833
$MOX_{MissActivity}$ Sedentary	193 (-1 to 386)	14.5 (-0.72 to 29.6)	18.5 (4.4 to 32.6)	-618 to 1003	0.91	0.036	874
$MOX_{Annegarn}$ Dynamic	273 (240 to 306)	64.3 (60.0 to 68.6)	64.3 (60.0 to 68.6)	137 to 410	0.41	0.070	551
$MOX_{Annegarn}$ Standing	-357 (-554 to -161)	-51.4 (-82.5 to -20.3)	61.5 (35.0 to 88.0)	-1178 to 466	0.62	0.004	1065
$MOX_{Annegarn}$ Sedentary	170 (-35 to 375)	13.2 (-2.3 to 28.7)	19.1 (5.0 to 33.1)	-687 to 1027	0.85	0.000	900
activPAL Dynamic[b]	-74 (-518 to 370)	-19.1 (-125.0 to 86.8)	75.4 (-22.0 to 172.7)	-646 to 1499	0.46	0.011	282
activPAL Standing[b]	-492 (-824 to -161)	-66.8 (-11.5 to -22.2)	71.2 (29.0 to 113.6)	-1667 to 692	0.55	0.003	1693
activPAL Sedentary[b]	421 (84 to 757)	21.0 (5.3 to 36.7)	21.0 (5.3 to 36.7)	-769 to 1610	0.28	0.001	1590
Fitbit Alta HR Dynamic[b]	347 (226 to 467)	81.8 (53.0 to 110.7)	96.6 (84.8 to 108.5)	-2854 to 555	0.05	0.000	831

[a] Gold standard minus Activity tracker.

[b] Five (5/20 25%) missing values for the activPAL and Fitbit Alta HR

Supplementary file 2. Sensitivity, specificity and accuracy by $MOX_{MissActivity}$ compared to the gold standard in comparison with reference activity trackers without outliers

**Behaviour**	**Sensitivity without outliers, mean (95% CI)**	**Specificity without outliers, mean (95% CI)**	**Accuracy without outliers, mean (95% CI)**
$MOX_{MissActivity}$ Dynamic	67.6 (61.7 – 73.4)	94.6 (93.3 – 95.9)	90.5 (88.9 – 92.2)
$MOX_{MissActivity}$ Standing	76.0 (66.1 – 86.0)	91.1 (86.0 – 96.3)	88.3 (85.0 – 91.6)
$MOX_{MissActivity}$ Sedentary	95.1 (89.7 – 100.0)	94.6 (87.9 – 100.0)	95.5 (92.3 – 98.6)
$MOX_{Annegarn}$ Dynamic	27.6 (23.7 – 31.5)	98.3 (97.7 – 99.0)	88.0 (86.4 – 89.6)
$MOX_{Annegarn}$ Standing	87.1 (76.4 – 97.7)	83.7 (78.0 – 89.3)	85.2 (81.9 – 88.5)
$MOX_{Annegarn}$ Sedentary	95.3 (89.6 – 100.0)	92.6 (83.2 – 100.0)	94.5 (91.3 – 98.6)
activPAL Dynamic[a]	44.3 (35.6 – 53.0)	95.0 (93.8 – 96.2)	86.9 (84.6 – 89.4)
activPAL Standing[a]	84.5 (81.5 – 87.6)	70.1 (57.3 – 82.9)	73.5 (63.4 – 83.6)
activPAL Sedentary[a]	76.5 (59.4 – 93.5)	98.4 (97.5 – 99.4)	85.0 (74.4 – 95.6)
Fitbit Alta HR Dynamic[a,b]	-	-	-

[a] Five (5/20 25%) missing values for the activPAL and Fitbit Alta HR

[b] Not possible to determine for Fitbit Alta HR, since Fitbit doesn’t provide raw data.
